# Difference in Visual Social Predispositions Between Newborns at Low- and High-risk for Autism

**DOI:** 10.1038/srep26395

**Published:** 2016-05-20

**Authors:** Elisa Di Giorgio, Elisa Frasnelli, Orsola Rosa Salva, Scattoni Maria Luisa, Maria Puopolo, Daniela Tosoni, NIDA-Network, Fabio Apicella, Antonella Gagliano, Andrea Guzzetta, Massimo Molteni, Antonio Persico, Giovanni Pioggia, Giovanni Valeri, Stefano Vicari, Francesca Simion, Giorgio Vallortigara

**Affiliations:** 1 CIMeC, Center for Mind/Brain Sciences, University of Trento, 38068, Italy; 2 Department of Psychology, College of Life and Environmental Sciences, University of Exeter, EX44QG, UK; 3 Department of Cell Biology and Neurosciences, Istituto Superiore di Sanità, 00161, Rome, Italy; 4 Department of Developmental and Social Psychology, University of Padova, 35135, Italy; 5 Cognitive Neuroscience Center, University of Padova, 35135, Italy; 6 Department of Developmental Neuropsychiatry, Scientific Institute "Fondazione Stella Maris", Viale del Tirreno 331, I-56018 Calambrone, Pisa, Italy; 7 Policlinico Universitario "G. Martino" di Messina, Italy; 8 Infant Neurology Section, Stella Maris Foundation, Pisa, Italy; 9 Scientific Institute IRCCS "E. Medea", Via Don Luigi Monza 20, 23842 Bosisio Parini, Italy; 10 Unit of Child and Adolescent NeuroPsychiatry, Laboratory of Molecular Psychiatry and Neurogenetics, University "Campus Bio-Medico", Rome, Italy; 11 Institute of Applied Sciences and Intelligent Systems, National Research Council of Italy (ISASI-CNR), Messina Unit, Italy; 12 Child Psychiatric Unit, IRCCS Ospedale Pediatrico Bambino Gesu, Rome, Italy

## Abstract

Some key behavioural traits of Autism Spectrum Disorders (ASD) have been hypothesized to be due to impairments in the early activation of subcortical orienting mechanisms, which in typical development bias newborns to orient to relevant social visual stimuli. A challenge to testing this hypothesis is that autism is usually not diagnosed until a child is at least 3 years old. Here, we circumvented this difficulty by studying for the very first time, the predispositions to pay attention to social stimuli in newborns with a high familial risk of autism. Results showed that visual preferences to social stimuli strikingly differed between high-risk and low-risk newborns. Significant predictors for high-risk newborns were obtained and an accurate biomarker was identified. The results revealed early behavioural characteristics of newborns with familial risk for ASD, allowing for a prospective approach to the emergence of autism in early infancy.

Autism spectrum disorders (ASD) compose an early-onset neurodevelopmental syndrome primarily characterized by impairments in social perception, cognition and communication^1^. In ASD, altered processing of social stimuli, such as faces^2^ and biological motion^3^, has been associated with impairments in the functioning of the “*social brain”*, a network of cortical areas mainly devoted to process social stimuli^4^. At present, the developmental origins of these abnormalities are unclear. According to one hypothesis—recently re-examined by one of its original proposers, Mark H. Johnson^5^—unusual development of the social brain in individuals with ASD may be due to an alteration and/or delay in the early activation of subcortical orienting mechanisms. In typical development, these mechanisms putatively bias newborns to orient to relevant social visual stimuli and simultaneously guide the specialization of cortical circuits devoted to detect and process social stimuli^6^,^7^.

This hypothesis was recently put into question by results from a recent study, which we will discuss in greater detail later, where infants as young as 2 months old (who were later diagnosed with ASD^8^) demonstrated normative levels of visual orienting to a social stimulus (i.e., to the area of the eyes within a face). Despite these results, visual orientation to social stimuli at even younger ages in infants at high-risk for autism has not been studied. Therefore, it is crucial to test newborns, as this may be the most suitable age that can provide insight into both normal and atypical activities of the subcortical orienting mechanisms. Here, we directly tested, for the very first time, the inborn predispositions to orient towards different classes of social and non-social stimuli in newborns at high familial risk for ASD (i.e., who have an older sibling diagnosed with ASD)^9^.

Typical newborns of several vertebrate species^10^, including humans^11^, come into the world equipped with inborn predispositions to preferentially attend to relevant social cues, such as faces^7^,^12^,^13^,^14^, direct eye-gaze^15^, and biological motion patterns^16^,^17^,^18^,^19^. These predispositions are thought to be mainly controlled by rapid and automatic subcortical orienting mechanisms, active at birth and present throughout the lifetime^20^. These mechanisms trigger the relevant visual input to developing cortical regions and contribute to the progressive specialization of the “*social brain*” network^6^,^21^. Therefore, if one considers these neonatal orienting mechanisms as precursors to the proper development of the social brain, it is plausible that their impairment could prevent newborns from focusing their attention on relevant social stimuli and could thus interfere with the typical specialization of the social brain, as observed in ASD.

Recent evidence, however, seems to contradict the innate social orienting mechanisms hypothesis. For example, one report has shown that a subcortical orienting mechanism is functional in autistic adults^22^. Furthermore, by the age of 7 months, both low- and high-risk ASD infants are able to attend to faces when presented among other objects^23^. Finally, early measures of eye-looking are reported at normative levels in 2-month-old infants later diagnosed with ASD, and only between 2 and 6 months of age the difference in looking at the eyes of others emerges, with typically-developing infants showing an increase in looking at the eyes of others during this period, whereas infants later diagnosed with ASD show a decrease in looking at the eyes of others. The authors of that study interpreted their findings as a demonstration that very early in life (at 2 months of age) infants later diagnosed with ASD and typically-developing infants share the same predispositions to pay attention to social stimuli^8^.

However, in all the above mentioned studies, subjects were either adults or infants as old as 2 months of age. Importantly, by this age, developing cortical areas already influence responses to social stimuli^6^. Moreover, 2-month-old babies have already had plenty of exposure to visual social stimuli, which may allow for learning-related compensatory mechanisms to occur. Indeed, Mark H. Johnson recently wrote: “*While the Shah et al. study*^22^ *suggests that the subcortical social orienting system is intact in adults with autism, it still remains possible that it is impaired or delayed around the time of birth, and this could have devastating knock-on consequences for the developing social brain network. To test this, we need direct evidence from young infants”*^5^. Therefore, data from newborns are needed to test the inborn social orienting mechanisms hypothesis: only with this age group can we directly investigate the activity of this mechanism without the interference of previous visual experience and other later-maturing mechanisms.

Accordingly, in the present paper we investigated whether the inborn predispositions to pay attention to multiple classes of social stimuli were different between groups of newborns at high-risk (HR) and low-risk (LR) for ASD. This comparison has already been employed in previous studies to identify relevant differences between HR and LR groups^8^,^23^,^24^. Specifically, we measured visual attention towards face-like stimuli^7^,^12^, images of real faces displaying direct eye-gaze^15^, and biological motion stimuli^18^,^19^ in HR and LR newborns. Previous studies demonstrated that typical newborns show a visual preference for (i.e., they looked longer at) the upright face-like pattern when contrasted with the inverted face-like pattern, the direct eye-gaze when contrasted with the averted eye-gaze and the biological motion stimuli when contrasted with both a random motion and a rigid motion. Typical newborns preferred the biological motion stimulus, a structured and a motion-coherent stimulus, compared to a random motion stimulus^18^, a non-structured and non-motion-coherent stimulus, as well as compared to a rigid motion stimulus, a structured but non-motion-coherent stimulus^19^. Therefore, we chose to employ these comparisons to test whether HR newborns are also sensitive to these characteristics of the biological motion.

We predicted that if some impairment in one of the mechanisms for social visual orienting is present from birth in infants at high-risk for ASD, then visual preferences for social stimuli should be absent or reversed and consistently lower in the high-risk group compared to the low-risk group. We can thus articulate the present study in three distinct, even though related, working hypotheses on the early phenotype of individuals at high-risk for ASD: selective impairment of i) the mechanism mediating orientation toward face-like configurations^7^; ii) the mechanism mediating orientation toward direct eye-gaze^25^; iii) the mechanism mediating orientation toward biological motion^26^. If our results confirm one or more of these hypotheses, it would suggest for the first time, that one or more of the subcortical orienting mechanisms mediating inborn predispositions for social stimuli might function differently in high-risk and low-risk newborns soon after birth.

## Results

We used a mobile lab to setup an infant-control visual preference procedure to test newborns in their homes. We presented four different visual preference tasks to each newborn. In each task, two stimuli were presented simultaneously: 1) an inverted face-like *vs.* an upright face-like pattern; 2) a real face displaying an averted gaze *vs.* a real face displaying a direct eye-gaze; 3) a rigid motion *vs.* a biological motion pattern; and 4) a random motion *vs.* a biological motion pattern. The visual behaviour of each participant in response to the stimuli presentation was video recorded and subsequently coded blind off-line by two independent coders. The dependent variables obtained from the videos were the percentage of the preference and the percentage of the number of fixations for each stimulus, and the direction of the first fixation (for details see Methods).

Differences between HR and LR newborns were analyzed using a mixed linear model with a fixed factor (HR *vs.* LR) and a random factor (coders). Independent significant predictors for HR (*vs.* LR) were obtained by logistic regression analysis for clustered data. A biomarker was derived from the estimated logit function and its diagnostic accuracy was assessed by ROC curve analyses^27^ (see Supplementary Information for details on statistical analyses).

**Figure 1:**
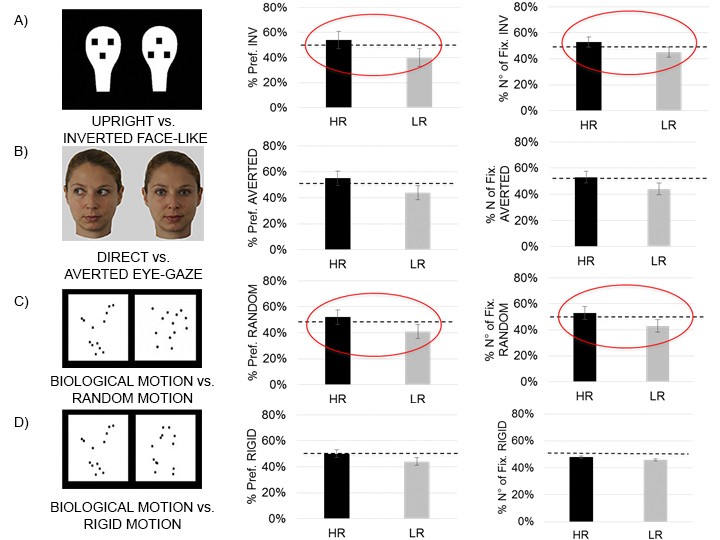
Stimuli and experimental results for high-risk (HR) and low-risk (LR) newborns. Each newborn was presented with four different visual preference tasks (**A–D**) in a random order. The main results for each group are expressed as the percentage of visual preference and percentage of the total number of fixations for a given stimulus (Mean ± SEM). Since we were interested in revealing predictors for the HR group, here we presented the percentage of preference and the percentage of the number of fixations towards the non-social stimuli as follows: (**A**) Inverted face-like pattern, (**B**) Averted eye-gaze, (**C**) Random motion pattern, and (**D**) Rigid motion pattern. Please note that (**B**) represents images similar to the stimuli used for the Adverted eye-gaze condition. Due to this Journal’s policy on permission for publication consent, it was not possible to publish the original stimuli. For the original stimuli please see Farroni *et al*.^15^. Significant differences between the two groups of participants are evident in the percentage of preference and number of fixations toward the inverted face-like pattern (*p* = 0.016 and p = 0.041 respectively), and in the percentage of preference and number of fixations toward the random motion pattern (*p* = 0.032 and p = 0.034 respectively) (all highlighted with red circles). Dashed lines indicate chance level (50%) (See also Supplementary Information).

Results obtained through the mixed model revealed that the percentage of the preference for the inverted *vs.* upright face-like pattern (*p* = 0.016), the random vs. biological motion pattern (p = 0.032), and the percentage of the number of fixations for the inverted vs. upright face-like pattern (p = 0.041) and for the random motion vs. biological motion pattern (p = 0.034) were significantly different between HR and LR newborns (Fig. 1). Specifically, what we found is that the HR group differed significantly from the LR group in the percentage of visual preference and number of fixations towards the inverted face-like pattern (i.e., HR looked longer at and did more fixations to the inverted face-like pattern *compared* to LR newborns), and in the percentage visual preference and number of fixations towards the random motion (i.e., HR newborns looked longer at and fixated a greater number of times towards the random motion stimulus *compared* to LR newborns).

Stepwise multivariate logistic regression analyses selected the percentage of preference for the inverted face-like pattern (*p* = 0.033) and the percentage of number of fixations toward random motion pattern (*p* = 0.007) as independent predictors for HR (*vs.* LR), supporting the efficacy of a multivariate approach to discriminate HR *vs.* LR.

**Figure 2:**
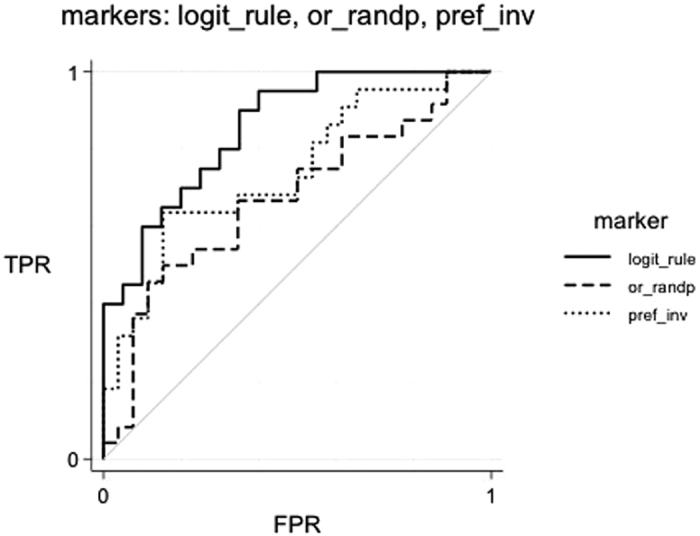
ROC curves for discriminating high-risk (HR) *vs.* low-risk (LR) newborns. ROC curves discriminate high-risk (HR) *vs.* low-risk (LR) newborns by different criteria: the percentage of visual preference for the inverted face-like pattern (Preference Inverted), the percentage of number of fixations toward the random motion pattern (Fixations random), and the criterion estimated from the logit function (Logit rule). AUC (Area under ROC curve): Preference inverted, 0.74; Fixations random, 0.72; Logit rule, 0.85. FPR = False Positive Rate; TPR = True Positive Rate.

The other variables, even if significantly different between HR and LR as shown by the mixed model, are not necessary because they do not add any further information. The estimated logit function rendered an accurate biomarker (Area under ROC curve, AUC: 0.85) (Fig. 2).

As for the analysis of the direction of the first fixation (dichotomized as Yes *vs.* No), when the raw data from each coder was evaluated separately, differences between HR and LR groups missed statistical significance, even if some qualitative differences in the HR and LR groups emerged (See TabS2, TabS3, TabS4 and TabS5 in Supplementary Information).

## Discussion

We demonstrate, for the first time, that inborn predispositions to attend to social stimuli, such as face-like patterns and biological motion, differ between HR and LR newborns. Specifically, we found that attention to these two kinds of non-social visual stimuli differs between HR and LR newborns. The main difference is due to the fact that HR newborns, compared to LR, were more interested in paying attention, in terms of both the percentage of visual preference and number of fixations, to the inverted face-like pattern and the random motion stimulus. These are striking findings because they provide the first evidence that impairments in the orienting mechanisms devoted to bias newborns’ visual attention to social stimuli are present very early in life in newborns at high-risk for autism.

Although a larger sample is needed to improve multivariate analyses, these findings provide an important contribution in expanding our current knowledge about the ontogeny of ASD and disclose early behavioural characteristics of infants with familial risk for ASD^28^,^29^.

Differences in the visual preference for the two kinds of social *vs.* non-social stimuli that we found in HR and LR newborns seem to contrast the hypothesis that all the subcortical orienting mechanisms are intact and typical in 2-month-old infants later diagnosed with ASD^8^. Indeed, the authors of that study interpreted their data as the demonstration that the subcortical social orienting mechanism toward faces must be intact from birth, and furthermore, that up to the age of 2 months ASD-babies would follow the typical developmental trajectory with a subsequent regression. A further conclusion derived by this picture was that in ASD the typical and normative transition from subcortical to cortical control of the visual social behaviour fails to occur.

However, from our point of view, this is not the only possible interpretation. Indeed, at two months of age we would expect the transition to cortical processing to have already occurred. In the well-known face preference model of typical development by Morton & Johnson^7^, the preferential orientation to faces at birth gradually declines around four to six weeks of life and re-emerges at about two months of age. In this model, the visual preference observed at birth is due to an automatic and reflexive subcortical orienting mechanism, whereas what takes over is described as a learning mechanism or experience-dependent mechanism. Moreover, behavioural findings on changes in visual preference during the first 2 months of age are connected to a neural shift from subcortical to cortical control that occurs at approximately 2 months of age. To sum up, the model proposes a U-shaped development for face perception in typical development, where at birth face preference is present and guided by a subcortical orienting mechanism (CONSPEC) and around two months of age it is guided by a cortical mechanism (CONLERN). Presumably then, around the age of 1.5 months these two mechanisms are in competition against one another and therefore face preference disappears. Thus, if the problem of ASD resides in the non-functional cortical mechanism, performance should be already atypical at that age.

In light of these considerations, Klin and colleagues^8^,^30^ suggested that the data for infants later diagnosed with ASD indicate a reflexive orientation that appears to persist beyond its developmentally appropriate window. In other words, according to their view, the reflexive subcortically-mediated orientation is not inhibited by the emergence of experience-dependent face or eye-fixation. The absence of a cortically-controlled, experience-dependent eye fixation curve in infants later diagnosed with ASD is suggested by the steady, continuing decline in their eye-fixation beginning at two months.

In the light of the complex developmental trajectory of normo-typical face-orienting, it is not possible to make inferences on the mechanisms active at birth based on data from older infants. So, while we agree with Klin’s suggestion^30^ on the presence of a decline in the cortically-controlled mechanism, we would like to propose another interpretation that is not only compatible with the data of Jones and Klin^8^, but also supported by our own results. We believe that in ASD at least some of the automatic orienting mechanisms are abnormal already at birth, as indicated by our data, causing an abnormal input to the developing cortical areas. Nevertheless, cortical areas involved in these kinds of social orienting develop all the same, based on maturational mechanisms, and they have an onset at about 2 months of age. This age could be marked by a peak of cortical function due to the initial activation of those areas (which could explain the data obtained in two-month-old infants, considering also the frequency with which eyes are seen by infants and the consequent learning effects). However, in the absence of a normally functioning subcortical mechanisms to support the activity of the developing cortical areas, their function would then decline, exactly as described by Klin and colleagues, resulting in an absent or atypical specialization. It is important to note that, in the model proposed by Johnson, subcortical mechanisms are supposed to be constantly active during all the lifespan of typically functioning individuals^20^. Even more importantly, the role of these orienting mechanisms would be also to interact with the developing cortical areas, possibly by neural signalling concomitant with the detection of appropriate social stimuli. This function could be crucial to the normal development of cortical mechanisms during the sensitive period in the first months of age, but also for their subsequent maintenance.

We are, nonetheless, aware of the limitations of the present work, in particular concerning the fact that validation in independent datasets and an adequate follow-up to assess the accuracy on a definite diagnosis (rather than consider newborns belonging to HR *vs.* LR groups) are still needed. Accuracy of the parameters in discriminating risk groups need to be validated. Although our findings suggest that risk groups differ in the selected parameters, assessment of its usefulness for the prediction of clinical diagnosis of autism is mandatory. Time is needed to allow participants to become older so that they can be properly tested to confirm (or reject) a clinical diagnosis of ASD. Despite the small sample size, observed patterns (statistically significant differences as well as not statistically significant differences) are statistically powerful (power > 0.90), except and for the percentage of number of fixations towards rigid motion vs. biological motion (0.10), highlighting the weakness of such visual preference task in screening for early behavioural indicators for ASD. Our project is ongoing and follow-up tests on these same participants at different age stages are currently underway, in line with the standard followed by previous studies^31^. We will correlate the data we have presented here with the results from follow-up studies and finally with the results from the clinical tests that all participants will be subject to starting at the age of 6 months until they will be 3 years old. Nevertheless, because our visual preference tasks are non-invasive and can be applied with ease, we believe that this study is a promising first step toward the creation of an early screening protocol for infants that may be considered as belonging to an at-risk-for-autism population. This protocol aims to promote early ASD screening, diagnosis and intervention during a time when the developing neural system is more plastic. From this point of view, it is plausible to consider visual attention to social and non-social stimuli as a possible early indicator of the presence of ASD symptoms, as already suggested in the literature^32^, but never tested prior to the present study in infants younger than 2 months old^30^.

Overall, these findings highlight the importance of investigating in further detail the role of the neonatal orienting mechanism in shaping the typical and atypical development of the social brain. Furthermore, our results underline how crucial it is to start monitoring infants at risk for ASD early in life.

## Methods

### Participants

Recruitment, ethical approval and informed consent were made available through the Italian network for early detection of Autism Spectrum Disorders (NIDA Network, www.progettonida.it). The Ethical Committee of the Istituto Superiore di Sanità (Rome, Italy) approved all the parts of the experimental protocol followed for the experiments described in this paper (approval code Istituto Superiore di Sanità CE/11/308). All methods were carried out in accordance with guidelines approved by the ethical Committee of the Istituto Superiore di Sanità in Rome (Italy) for this project.

Pregnant mothers of low-risk (LR) and high-risk (HR) (i.e., younger siblings of ASD probands) newborns were enrolled from various regions throughout Italy and invited to attend multiple research visits until their children reach 36 months of age. The exclusion criteria for both low-risk and high-risk newborns included prematurity, low birth weight, Apgar score <7, and presence of other medical, genetic or neurological conditions. Newborns were tested at home only if awake and in an alert state, and after the parents had provided informed consent.

#### High-risk (HR) newborn group

A total of 17 (7 males) healthy and full-term newborns from NIDA Network took part in the current study and were tested in the four visual preference tasks. Data from 4 newborns (1 male) were discarded because they started to cry at the beginning of the experimental session and as a consequence, it was impossible to collect high-quality data (N = 2) or because other medical and neurological conditions were diagnosed after recruitment, including visual impairment (N = 2). Therefore the final sample consisted of 13 newborns (6 males). Their postnatal age ranged from the 6^th^ to the 10^th^ day (range: 137 to 248 h). All participants met the screening criteria adopted by the guidelines proposed by the NIDA network: newborns had an older brother or sister who had received a clinical diagnosis of an autism spectrum disorder from expert clinicians (N = 9, sibling had ASD and an accompanying intellectual impairment; N = 4, sibling had ASD without any accompanying intellectual impairment), had a birth weight between 2810 and 3840 g (*M* = 3290 g, *SD* = 356.3), and had an Apgar score between 8 and 10 at the 5^th^ minute.

#### Low-risk (LR) newborn group

A total of 17 (8 males) newborns were tested in the four visual preference tasks and all of them were part of the final sample. Data from a newborn (female) were discarded because she started to cry at the beginning of the experimental session and as a consequence, it was impossible to collect high-quality data (N = 1). Therefore, the final sample comprised 16 newborns (8 males). Their postnatal age ranged from the 6^th^ to the 10^th^ day (range: 145 to 265 h). Inclusion criteria included the full-term birth (after the 37^th^ gestational week), normal birth weight between 2800 and 3980 g (*M* = 3299 g, *SD* = 344.9), Apgar score between 9 and 10 at the 5^th^ minute and, crucially, none of them had first- or second-degree relatives with autism, as confirmed through interviewing the parents regarding family medical history.

Newborns in both groups were tested with all the four visual preference tasks, but some babies did not complete all the tasks due to fussiness. Therefore, for each of the visual preference tasks, we have different sample sizes. The final sample sizes for each task were as follows: face-like visual preference task, N = 11 HR and N = 13 LR newborns; eye-gaze visual preference task, N = 12 HR and N = 14 LR newborns; biological motion vs. random motion visual preference task, N = 12 HR and N = 13 LR newborns; biological motion vs. rigid motion visual preference task, N = 10 HR and N = 10 LR newborns.

### Stimuli

#### Inverted face-like vs. upright face-like pattern

These stimuli were those used by Valenza and colleagues^12^, and consisted of two head-shaped two-dimensional white forms, 22 × 15 cm (36° × 27°), with three black squares (2.5 × 2.5 cm, 4.8° × 4.8°). The black squares were placed in the appropriate location for the eyes and the mouth in the upright face-like pattern resembling a schematic human face, whereas they were rotated 180° on the vertical axis in the inverted face-like pattern. The distance between the two stimuli on the screen was 10 cm (19°).

#### Averted vs. direct eye-gaze

The stimuli were those used by Farroni *et al*.^15^ and consisted of coloured images of real female faces directing their gaze straight-on to the observer (direct eye-gaze) or averted to one side (averted eye-gaze). The stimuli were 19.5 × 15.5 cm (33° × 27°). The distance between the two stimuli on the screen was 10 cm (19°).

#### Random motion vs. biological motion pattern

The biological motion, random motion and rigid motion pattern stimuli were the same used in our previous studies^18^,^19^. The biological motion stimulus consisted of a pattern of 13 moving black squares on a white background representing a walking hen, whereas the same pattern of squares moving randomly constituted the non-biological random motion stimulus^18^. The dimensions of the stimuli were 14.5 × 16.5 cm (26° × 29°) and each black square measured 0.6 × 0.6 cm (1° × 1°) on the screen. The distance between the two stimuli on the screen was 9.5 cm (18°).

#### Rigid motion vs. biological motion pattern

The biological motion pattern (walking hen) was contrasted with a non-biological, rigid motion pattern, created by rotating the first frame of the hen animation around its vertical axis^19^. The dimensions of the stimuli and the distance between them were the same as those of the previous pair.

### Apparatus and procedure

The apparatus and procedure were the same for all the four visual preference tasks. Newborns were tested at home with a mobile lab that included a computer monitor (Dell Display Flat Panel 27”) where the stimuli were presented to the newborn, a laptop to run the stimuli presentation (HP Elitebook 8570P, E-prime 2.0 software), a high-resolution video camera (JVC TK-C750 U) placed above the monitor to record the eye movements of the newborn while attending to the stimuli presentation, a mixer (Datavideo HS-600) to assemble the stimuli from the laptop with the output of the video camera, and a video recorder to save all the data. An infant-control preferential looking technique, in which each trial ended when the newborn stopped fixating at the display for at least 10 s, was used for all the four tasks. For each task, the two visual stimuli were simultaneously presented to the newborns on the computer monitor (refresh rate = 60 Hz, 1240 × 768). Indeed, for all the four tasks, two trials were administered and the relative left/right position of each stimulus in the pair was counterbalanced. Importantly, all newborns were presented with all the four tasks and the order of presentation was randomized among participants. The baby sat on an experimenter’s lap at a distance of about 30 cm in front of the monitor. Above the monitor, the video camera recorded the eye movements of the newborns to monitor their looking behaviour on-line and to allow off-line coding of their fixations. At the beginning of both the preference test trials, a red disc was shown on a black background to attract the newborn’s gaze to the centre of the monitor. The disc grew and then shrank back continuously, from a size of 1.8 cm to a size of 2.5 cm. As soon as the newborn’s gaze was properly aligned with the red disc, a second experimenter monitoring on-line the newborn’s visual attention through the video camera started the sequence of trials by pressing a key on the keyboard that automatically turned off the central disc and activated the onset of the stimuli.

The dependent variables that we measured, as in previous studies^12^,^33^, were some of those suggested by Cohen’s model of attention^34^ as indexes of the attention-getting (orienting) and of the attention-holding (detecting) mechanisms. Therefore, as indexes of attention-getting mechanisms, the coders recorded, separately for each stimulus and each position, the direction of the first fixation and the number of fixations (or orienting responses) for the two stimuli. These measures was converted to a percentage score by computing the total number of fixations towards a given stimulus divided by the total number of fixations towards both stimuli in each visual preference condition, X100. As indexes of attention-holding mechanisms, the coders calculated the percentage of visual preference, that is, the length of time for which each newborn looked at the non-social (or at the social stimulus) divided by the total time spent looking at both stimuli in each test trial. As for the first measure, the score was then multiplied by 100. Therefore, scores significantly above or below 50% indicate a visual preference for the non-social or for the social stimulus in each of the four visual preference tasks. In the present study, for the convenience of explanation, we calculated the percentage index using the non-social stimulus of each pair (inverted face, adverted gaze, rigid and random motion) as a reference point (the first term of the equation, the dividend). Thus, values above chance level indicate a higher attendance for the non-social stimulus, and vice versa (see also Supplementary Information).

Videotapes of newborns’ eye movements, throughout the two trials of each test, were subsequently coded off-line by two different coders, unaware of the group to which each newborn belonged (HR *vs.* LR) and of the stimuli presented.

## Additional Information

**How to cite this article**: Di Giorgio, E. *et al*. Difference in Visual Social Predispositions Between Newborns at Low- and High-risk for Autism. *Sci. Rep.* **6**, 26395; doi: 10.1038/srep26395 (2016).

## Supplementary Material

Supplementary Information
