# Corrigendum: Difference in Visual Social Predispositions Between Newborns at Low- and High-risk for Autism

**DOI:** 10.1038/srep29860

**Published:** 2016-07-18

**Authors:** Elisa Di Giorgio, Elisa Frasnelli, Orsola Rosa Salva, Maria Luisa Scattoni, Maria Puopolo, Daniela Tosoni, NIDA-Network, Fabio Apicella, Antonella Gagliano, Andrea Guzzetta, Massimo Molteni, Antonio Persico, Giovanni Pioggia, Giovanni Valeri, Stefano Vicari, Francesca Simion, Giorgio Vallortigara

Scientific Reports 6: Article number: 2639510.1038/srep26395; published online: 05202016; updated: 07182016

The original version of this Article contained an error in the spelling of the author Maria Luisa Scattoni which was incorrectly given as Scattoni Maria Luisa. These errors have now been corrected in the PDF and HTML versions of the Article.

