# Supplementary material for: Difference in Visual Social Predispositions Between Newborns at Low- and High-risk for Autism
Source: Sci Rep. 2016 May 20;6:26395. doi: 10.1038/srep26395 (PMC4873740; doi:10.1038/srep26395)
Supplement: Supplementary Information [file srep26395-s1.pdf]

Supplementary Information

"Difference in Visual Social Predispositions Between Newborns at Low- and High-risk for Autism"

Elisa Di Giorgio, Elisa Frasnelli, Orsola Rosa Salva, Maria Luisa Scattoni, Maria Puopolo, Daniela Tosoni, NIDA-Network, Francesca Simion and Giorgio Vallortigara.

Statistical Analyses

For each task, the differences between HR and LR groups in the percentage of visual preference and percentage of number of fixations for non-social *vs.* social stimuli were analyzed with a mixed linear model with a fixed factor (HR *vs.* LR) and a random factor (coders). Logistic regression was used with the aim to predict the group membership of the participants (HR *vs.* LR) based on collected variables. Univariate analyses were followed by stepwise multivariate analysis to select independent significant predictors for HR (*vs.* LR). To account for dependency of data from the same subject, the cluster option was used to obtain robust estimates. A multivariate biomarker was derived from the estimated logit function. ROC curve analyses were performed and the area under ROC curve (AUC) was calculated to assess diagnostic accuracy. With regards to the direction of the first fixation, successes in each trial (1 and 2), defined as the event in which the first fixation was toward the social stimuli in each of the four visual preference tasks, were summed. LR and HR groups were compared in terms of presence/absence of at least one success (sum of the two test trials >=1) by logistic regression with clustering to account for dependency of data from the same subject by two coders. Fisher's exact test was used for comparisons of groups on categorical variables. STATA 13 was used for statistical analyses.

T-test Analyses on Each of the Four Visual Preference Tasks for Both Groups

We conducted additional analyses on preference scores (percentages) for the non-social visual stimulus in each visual preference task, analyses routinely performed with data from newborns. As stated before, the length of time each newborn looked at the non-social stimulus (or at the social stimulus) was divided by the total time spent looking at both test stimuli. The score was then multiplied by 100. Therefore, scores significantly below or above 50% indicate a visual preference for the social (or for the non-social stimulus).

It appears evident that HR did not show a visual preference for the non-social stimulus, pointing to differences between HR and LR newborns when presented with social and non-social stimuli. Here, we report the statistical significance of the t-test for each of the four visual preference tasks for both groups divided by the two coders (Coder 1 and Coder 2).

Percentage of Visual Preference

Percentage of Visual Preference for the Inverted face-like pattern:

CODER 1: HR (M = 54%, SD = 13), t (10) = .96, p = .36, n.s.
LR (M = 40%, SD = 11), t (12) = 3.5, p = *.005.*
CODER 2: HR (M = 53%, SD = 15), t (10) = .63, p = .54, n.s.
LR (M = 40%, SD = 13), t (12) = 2.8, p = *.035.*
Inter-coder correlation HR, r =.89
Inter-coder correlation LR, r =.82

Percentage of Visual Preference for the Averted eye-gaze:

CODER 1: HR (M = 52%, SD = 15), t (11) = .46, p = .66, n.s.
LR (M = 44%, SD = 9), t (13) = 2.6, p = *.024.*
CODER 2: HR (M = 53%, SD = 16), t (11) = .42, p = .68, n.s.
LR (M = 43%, SD = 14), t (13) = 1.8, p = *.10, n.s.*
Inter-coder correlation HR*, r =.86*
Inter-coder correlation LR*, r =.88*

Percentage of Visual Preference for the Random motion:

CODER 1: HR (M = 51%, SD = 13), t (11) = .28, p = .78, n.s.
LR (M = 41%, SD = 11), t (12) = 3.1, p = *.009.*
CODER 2: HR (M = 54%, SD = 13), t (11) = 1.0, p = .33, n.s.
LR (M = 45%, SD = 144), t (12) = 1.2, p = .24, n.s.
Inter-coder correlation HR*, r =.76*
Inter-coder correlation LR*, r =.87*

Percentage of Visual Preference for the Rigid motion:

CODER 1: HR (*M* = 51%, *SD* = 9), *t* (9) = .29, *p* = .78, n.s.
LR (*M* = 44%, *SD* = 10), *t* (9) = 1.8, *p* = .10, n.s.
CODER 2: HR (*M* = 48%, *SD* = 11), *t* (9) = .50, *p* = .63, n.s.
LR (*M* = 45%, *SD* = 13), *t* (9) = 1.3, *p* = .23, n.s.
Inter-coder correlation HR*, r =.94*
Inter-coder correlation LR*, r =.87*

Percentage of Number of Fixations

Percentage of Number of Fixations for the Inverted face-like pattern:

CODER 1: HR (*M* = 55%, *SD* = 10), *t* (10) = 1.9, *p* = .09, n.s.
LR (*M* = 45%, *SD* = 7), *t* (12) = 2.4, p = .035.
CODER 2: HR (*M* = 52%, *SD* = 11), *t* (10) = .54, *p* = .60, n.s.
LR (*M* = 47%, *SD* = 9), *t* (12) = 1.2, p = .27, n.s.
Inter-coder correlation HR, *r =.51*
Inter-coder correlation LR, *r =.51*

Percentage of Number of Fixations for the Averted eye-gaze:

CODER 1: HR (*M* = 52%, *SD* = 13), *t* (11) = .47, *p* = .64, n.s.
LR (*M* = 44%, *SD* = 9), *t* (13) = 2.3, *p = .040.*
CODER 2: HR (*M* = 51%, *SD* = 13), *t* (11) = .18, *p* = .86, n.s.
LR (*M* = 52%, *SD* = 10), *t* (13) = .83, p = .42, n.s.
Inter-coder correlation HR, *r =.86*
Inter-coder correlation LR, *r =.55*

Percentage of Number of Fixations for the Random motion:

CODER 1: HR (*M* = 52%, *SD* = 9), *t* (11) = .94, *p* = .37, n.s.
LR (*M* = 43%, *SD* = 10), *t* (12) = 2.6, *p* = .024.
CODER 2: HR (*M* = 53%, *SD* = 11), *t* (11) = .95, *p* = .36, n.s.
LR (*M* = 49%, *SD* = 7), *t* (12) = .67, *p* = .51, n.s.
Inter-coder correlation HR, *r =.77*
Inter-coder correlation LR, *r =.49*

Percentage of Number of Fixations for the Rigid motion:

CODER 1: HR (*M* = 49%, *SD* = 8), *t* (9) = .59, *p* = .57, n.s.
LR (*M* = 46%, *SD* = 8), *t* (9) = 1.7, *p* = .13, n.s.
CODER 2: HR (*M* = 48%, *SD* = 8), *t* (9) = .65, *p* = .53, n.s.
LR (*M* = 48%, *SD* = 12), *t* (9) = .49, *p* = .63, n.s.
Inter-coder correlation HR, *r =.92*
Inter-coder correlation LR, *r =.67*

Supplementary Table S1

Coder 1	Inverted Face-like pattern				Averted Eye-Gaze				Random Motion				Rigid Motion			
	% Preference		% Number of fixations		% Preference		% Number of fixations		% Preference		% Number of fixations		% Preference		% Number of fixations	
	Mean	Dev. st	Mean	Dev. St	Mean	Dev. st	Mean	Dev. St	Mean	Dev. st	Mean	Dev. St	Mean	Dev. st	Mean	Dev. St
HR	54%	13%	55%	10%	52%	15%	52%	13%	51%	13%	52%	9%	51%	9%	49%	8%
LR	40%	11%	45%	7%	44%	9%	44%	9%	41%	11%	43%	10%	44%	10%	46%	8%

Coder 2	Inverted Face-like pattern				Averted Eye-Gaze				Random Motion				Rigid Motion			
	% Preference		% Number of fixations		% Preference		% Number of fixations		% Preference		% Number of fixations		% Preference		% Number of fixations	
	Mean	Dev. st	Mean	Dev. St	Mean	Dev. st	Mean	Dev. St	Mean	Dev. st	Mean	Dev. St	Mean	Dev. st	Mean	Dev. St
HR	53%	15%	52%	11%	53%	16%	51%	13%	54%	13%	53%	11%	48%	11%	48%	8%
LR	40%	13%	47%	9%	43%	14%	52%	10%	45%	14%	49%	7%	45%	13%	48%	12%

Table S1. Raw data for all visual preference tasks with means and standard deviations summarized at the group level for both coders. Percentage of visual preference and percentage of the number of fixations are reported.

Supplementary Table S2

CODER 1	Sum of First Fixations to Upright face-like	
	**0** (Failure NO)	**1&2** (Success = YES)
**HR**	5 (45%)	6 (55%)
**LR**	2 (15%)	11 (85%)

Fisher's $p = 0.182$

CODER 2	Sum of First Fixations to Upright face-like	
	**0** (Failure = NO)	**1&2** (Success = YES)
**HR**	5 (45%)	6 (55%)
**LR**	2 (15%)	11 (85%)

Fisher's $p = 0.182$

TabS2: Distribution of the first fixations towards the Upright face-like stimulus of High-Risk (HR) and Low-Risk (LR) newborns in the evaluation by two independent coders.

Supplementary Table S3

CODER 1	Sum of First Fixations to Direct Eye-Gaze	
	**0** (Failure NO)	**1&2** (Success = YES)
**HR**	2 (17%)	10 (83%)
**LR**	1 (7%)	13 (93%)

Fisher's $p = 0.580$

CODER 2	Sum of First Fixations to Direct Eye-Gaze	
	**0** (Failure = NO)	**1&2** (Success = YES)
**HR**	3 (25%)	9 (75%)
**LR**	1 (7%)	13 (93%)

Fisher's $p = 0.306$

TabS3: Distribution of the first fixations towards the Direct eye-gaze stimulus of High-Risk (HR) and Low-Risk (LR) newborns in the evaluation by two independent coders.

Supplementary Table S4

CODER 1	Sum of First Fixations to Bio Motion vs. Random Motion	
	**0** (Failure NO)	**1&2** (Success = YES)
**HR**	4 (33%)	8 (67%)
**LR**	2 (15%)	11 (85%)

Fisher's *p* = 0.378

CODER 2	Sum of First Fixations to Bio Motion vs. Random Motion	
	**0** (Failure = NO)	**1&2** (Success = YES)
**HR**	3 (25%)	9 (75%)
**LR**	1 (8%)	12 (92%)

Fisher's *p* = 0.322

TabS4: Distribution of the first fixations towards the Random Motion stimulus of High-Risk (HR) and Low-Risk (LR) newborns in the evaluation by two independent coders.

Supplementary Table S5

CODER 1	Sum of First Fixations to Bio Motion vs. Rigid Motion	
	**0** (Failure NO)	**1&2** (Success = YES)
**HR**	3 (30%)	7 (70%)
**LR**	3 (30%)	7 (70%)

Fisher's *p* = 1.000

CODER 2	Sum of First Fixations to Bio Motion vs. Rigid Motion	
	**0** (Failure = NO)	**1&2** (Success = YES)
**HR**	3 (30%)	7 (70%)
**LR**	2 (20%)	8 (80%)

Fisher's *p* = 1.000

TabS5: Distribution of the first fixations towards the Rigid Motion stimulus of High-Risk (HR) and Low-Risk (LR) newborns in the evaluation by two independent coders.
